# A Method for Multiple Mycotoxin Analysis in Wines by Solid Phase Extraction and Multifunctional Cartridge Purification, and Ultra-High-Performance Liquid Chromatography Coupled to Tandem Mass Spectrometry

**DOI:** 10.3390/toxins4060476

**Published:** 2012-06-15

**Authors:** Masayoshi Tamura, Ayumi Takahashi, Atsuo Uyama, Naoki Mochizuki

**Affiliations:** Research Laboratories for Food Safety Chemistry, Asahi Group Holdings, Ltd., 1-21, Midori 1, Moriya, Ibaraki 302-0106, Japan; Email: masayoshi.tamura@asahigroup-holdings.com (M.T.); ayumi.takahashi@asahigroup-holdings.com (A.T.); atsuo.uyama@asahigroup-holdings.com (A.U.)

**Keywords:** UHPLC-MS/MS, mycotoxins, wine, solid phase extraction, multiple analyses

## Abstract

An analytical method using two solid phase extractions and ultra-high-performance liquid chromatography coupled to tandem mass spectrometry (UHPLC-MS/MS) was developed for the identification and quantification of 14 mycotoxins (patulin, deoxynivalenol, aflatoxins B_1_, B_2_, G_1_, G_2_, M_1_, T-2 toxin, HT-2 toxin, zearalenone, fumonisins B_1_, B_2_, B_3_, and ochratoxin A) in domestic and imported wines. Mycotoxins were purified with an Oasis HLB cartridge, followed by a MultiSep^TM^ #229 Ochra. As a result, sufficient removal of the pigments and highly polar matrices from the red wines was achieved. UHPLC conditions were optimized, and 14 mycotoxins were separated in a total of 13 min. Determinations performed using this method produced high correlation coefficients for the 14 mycotoxins (R > 0.990) and recovery rates ranging from 76 to 105% with good repeatability (relative standard deviation RSD < 12%). Twenty-seven samples of domestic and imported wines were analyzed using this method. Although ochratoxin A (OTA) and fumonisins (FMs) were detected in several samples, the FM levels were less than limits of quantification (LOQs) (1 μg/L), and even the largest of the OTA levels was below the EU regulatory level (2 μg/L). These results suggest that the health risk posed to consumers from the wines available in Japan is relatively low.

## 1. Introduction

Mycotoxins are secondary metabolites produced by several species of fungi mainly belonging to the genera *Aspergillus*, *Penicillium*, and *Fusarium*, and are found in several kinds of food, especially cereals and cereal products. Mycotoxins are associated with carcinogenic, teratogenic, nephrotoxic, and hepatotoxic properties, which cause severe health effects and pose serious problems to food safety worldwide. 

According to research by the Codex Alimentarius Commission in 1998, it has been reported that wine is the second largest source of total ochratoxin A (OTA) intake in Europe after cereals [1]. OTA has been reported to be nephrotoxic and carcinogenic to both humans and animals, and a provisional tolerable weekly intake (PMTWI) of 100 ng/kg of body weight (kg bw)/week has been established by the Joint FAO/WHO Expert Committee on Food Additives (JECFA) [2]. This is the lowest regulatory level for mycotoxins with the exception of aflatoxin, for which a tolerable intake is not established, because of their genotoxic effects. The occurrence of OTA in wine has been reported in several studies [3,4,5,6,7], and several countries have their own regulations to control OTA in wine products; for instance, its maximum level established by the European Union (EU) is 2 μg/L [8]. A recent study also reports the occurrence of fumonisins (FMs), in particular FMB2, in red wine [9]. FMs have been shown to be carcinogenic and cytotoxic [10,11]. They contaminate corn and corn-based foods and feeds at high-concentration and high-frequency, being widespread around the world [7,12,13,14,15,16] The JECFA reported that a provisional maximum tolerable daily intake (PMTDI) is 2 μg/kg bw/day for FMB1, B2, and B3, alone or in combination [2]. Whereas FMs were thought until recently to occur only from *Fusariumverticillioides* and *F. proliferatum*, *Aspergillus niger* (*A. niger*), which is an OTA producer known to damage many crops and foods worldwide, was found capable of producing FMs [17,18] This indicates that FMs may be present in a wider variety of commodities and that both OTA and FMs contamination should be of concern. Additionally, Tabata reported that patulin (PAT) can also contaminate grapes [19]. It is produced by several species of fungi in the genera *Aspergillus* and *Penicillium* and is known as an apple contaminant. PAT has been reported to cause gastrointestinal and kidney dysfunction, and the JECFA established the PMTDI level of 0.4 μg/kg bw/day [20]. Thereby, the Codex instituted that the maximum permitted level of PAT in apples or apple juice is 50 μg/kg [21]. Along with these international trends, the Ministry of Health, Labour and Welfare (MHLW) also set the maximum level of 50 μg/kg in apple juice in Japan [22]. In contrast, no regulation on PAT has been provided in grapes and wines. Tabata reported that PAT was detected at high levels in the grapes inoculated with *P. expansum* and suggested potential risk of PAT contamination in grapes. It was also detected in the samples labeled as 100% Japan’s domestically-produced grape juice [19].

As described above, it has been clear that the mycotoxins have been able to contaminate wines. Although the detection of fusarium mycotoxins (NIV, DON, HT-2, T-2, ZON) and AFs have not been reported in wine, this is not assurance that they do not contaminate wines and wine-materials. Especially concerening aflatoxins, AFB1 has only been detected from crops, but in late years, the fungi producing AFs have changed under the influence of global warming, and the detection of AFB2 and G2 have increased. Therefore each country has strengthened regulation of AFs. These facts suggest that wine can be contaminated with mycotoxins for which the levels have not been legally controlled; hence it is essential to establish procedures for simultaneously determining multiple mycotoxins in wine. The MHLW also stipulated that the maximum level of total aflatoxins (AFs) is 10 μg/kg in all foods, and this has been applied in Japan since October 1, 2011 [23]. However, it will be time-consuming to separately determine mycotoxin levels in wine products.

**Figure 1:**
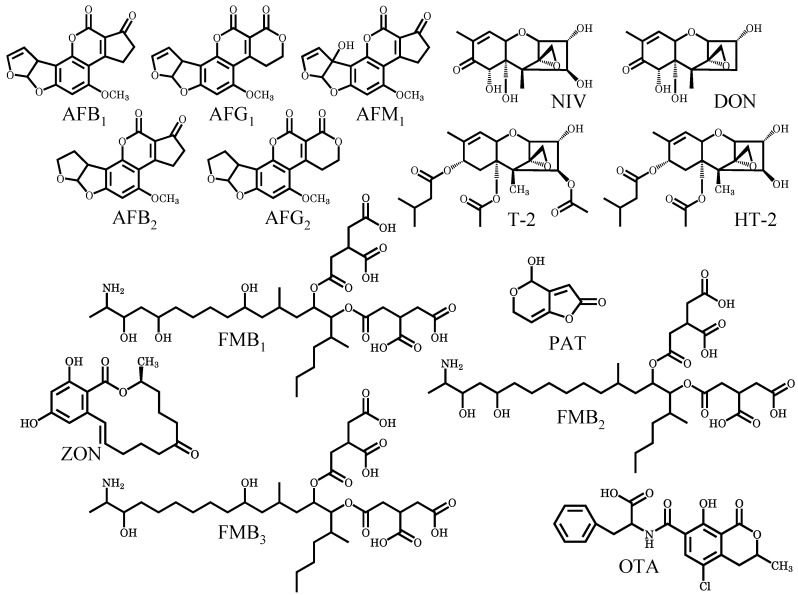
Structures of target mycotoxins.

While the mycotoxin regulations are being strengthened worldwide, we developed an accurate and sensitive method for the analysis of multiple mycotoxins in beer-based drinks, and this method shortened the time required for the assay [24]. In order to expand this multiple mycotoxin analysis, we considered to applying this method to foods, food-materials, and other alcoholic drinks. In this study, one of the alcoholic drinks, wine, was selected to use as analytical samples to include many matrices. This study thus aimed to develop a method for the high-throughput analysis of multiple mycotoxins in wine. The method was then applied to quantitatively determine mycotoxins in domestic and imported products. The fourteen mycotoxins that are attracting global attention due to significant toxicities were selected for the target analytes (Figure 1). 

## 2. Materials and Methods

### 2.1. Samples

Fourteen and thirteen samples of red and white wines, respectively, were randomly obtained from local supermarkets in Japan in 2010. All samples were then stored in a refrigerator until analysis.

### 2.2. Chemicals and Reagents

The following reagents were purchased from Kanto Chemical Co., Inc. (Tokyo, Japan): methanol of LC/MS grade; ammonium acetate, formic acid, and acetic acid of guaranteed reagent grade; and acetonitrile of LC/MS grade and that of pesticide residue and polychlorinated biphenyl (PCB) analytical grade. The acetonitrile of LC/MS grade was used for preparation of the working solutions and UHPLC-MS/MS analysis, and that of pesticide residue and PCB analytical grade was for sample preparation. Water was purified with a Millipore (Molsheim, France) Milli-Q system. An Oasis HLB cartridge (200 mg/6 mL) was purchased from Waters (Manchester, UK). A MultiSep^TM^ #229 Ochra cartridge was from Romer Labs (Bukit Merah, Singapore). AFB1, AFB2, AFG1, AFG2 (each at 3 μg/mL), and AFM1 (10 μg/mL) standard solutions were obtained from Sigma-Aldrich (St. Louis, MO). PAT, ZON (each at 100 μg/mL), FMB1, B2, and B3 (each at 50 μg/mL) standard solutions were from Biopure (Tulln, Austria). NIV, DON, HT-2 toxin (HT-2), and T-2 toxin (T-2) (each at 100 μg/mL) standard solutions were from Wako Pure Chemical Ind., Ltd. (Osaka, Japan). The working solutions were prepared as follows: FMs solution containing FMB1, B2 and B3 (each at 5 μg/mL) was diluted in acetonitrile/water (50/50 v/v) and stored in a refrigerator; AFs solution containing AFB1, B2, G1, G2, and M1 (each at 1 μg/mL), OTA solution (1 μg/mL), and the other mycotoxins solution containing PAT, NIV, DON, ZON (each at 10 μg/mL), HT-2, and T-2 (each at 2 μg/mL) were diluted in acetonitrile and were stored in a freezer.

### 2.3. Sample Preparation

A 5-mL sample of wine and 25 mL of 10 mM ammonium acetate aqueous solution were added into a 50-mL polypropylene centrifuge tube and were then mixed. The mixture was applied to an Oasis HLB cartridge previously conditioned with 5 mL of acetonitrile and 5 mL of 10 mM ammonium acetate aqueous solution. The cartridge was washed with 5 mL of 10 mM ammonium acetate aqueous solution. The mycotoxins retained in the cartridge were eluted with 5 mL of 10 mM ammonium acetate aqueous solution/acetonitrile (1/1 v/v) and then with 5 mL of acetonitrile. The eluate was mixed and evaporated to dryness at 40 °C under a nitrogen stream. The dried sample was dissolved with 1 mL of water. Subsequently 60 μL of formic acid and 5 mL of acetonitrile were added to the sample, and they were mixed together. The mixture was applied to a MultiSep #229 Ochra cartridge. Four mL of the purified eluate was evaporated to dryness at 40 °C under a nitrogen stream and was dissolved with 500 μL of 10 mM ammonium acetate aqueous solution/acetonitrile (85/15 v/v). Each sample was filtrated through a 0.20 μm PTFE filter immediately before UHPLC-MS/MS analysis.

### 2.4. UHPLC-MS/MS Analysis

An UHPLC-MS/MS analysis was performed using an ACQUITY UPLC^TM^ system coupled to a Quattro Premier^TM^ XE tandem quadrupole mass spectrometer (Waters, Manchester, UK). MassLynx 4.1 software equipped with QuanLynx software (Waters) was used to control the instruments and to process the data. An ACQUITY UPLC consisting of a binary pump, an auto-sampler, and a column heater was used. Chromatographic separation was carried out on an ACQUITY UPLC BEH C18 (1.7 μm, 2.1 × 100 mm; Waters). Solvent A was water, and solvent B was 2% acetic acid and 0.1mM ammonium acetate in methanol. In the previous study [24], it was clear that 2 gradient conditions were needed in multiple mycotoxin analysis because the carry-over of FMs and OTA were observed. The two gradient profiles were performed as follows: 0–1 min (5% B); 8 min (80% B), and 8.01–10 min (5% B) for the mycotoxins except for FMB1, FMB2, FMB3, and OTA: 0 min (55% B), 5 min (80% B), and 5.01–7 min (55% B) for FMB1, FMB2, FMB3, and OTA. The flow rate was set at 0.3 mL/min and the column temperature was 40°C. An auto-sampler was used to inject 5 μL.

A Quattro Premier^TM^ XE tandem quadrupole mass spectrometer was operated in both positive and negative mode with the electrospray-ionization (ESI) source. The operating parameters were optimized under the following conditions: capillary voltage, 3.0 kV (positive and negative mode); ion source temperature, 120 °C; desolvation temperature, 450 °C; cone gas flow, 50 L/h; desolvation gas flow, 800 L/h (both gases were nitrogen); and collision gas flow, 0.3 mL/min (argon gas). Multiple reaction monitoring (MRM) transitions, applied cone voltages, and collision energies are summarized in Table 1.

**Table 1:** MS/MS conditions for the determination of 14 mycotoxins.

Mycotoxin	Polarity	Cone voltage (V)	Precursor ion (*m/z*)	Quantification ion		Identification ion	
				Collision energy (eV)	Product ion(*m/z*)	Collision energy (eV)	Product ion(*m/z*)
PAT	ESI-	20	153	7	109	7	135
DON	ESI+	27	297	13	249	13	231
AFG2	ESI+	53	331	25	313	30	245
AFM1	ESI+	40	329	25	273	40	229
AFG1	ESI+	53	329	27	243	23	311
AFB2	ESI+	50	315	27	287	30	259
AFB1	ESI+	53	313	23	285	37	241
HT-2	ESI+	15	442	10	263	15	215
T-2	ESI+	20	484	13	245	23	185
ZON	ESI-	48	317	25	175	20	273
FMB1	ESI+	57	722	45	334	40	352
FMB2	ESI+	57	706	37	336	40	318
FMB3	ESI+	57	706	37	336	40	318
OTA	ESI+	30	404	23	239	15	358

### 2.5. Performance Evaluation

Because no authorized guideline for the analysis of multiple mycotoxins has been issued, we consulted “about the total aflatoxins analysis” provided by the Japanese Ministry of Health, Labour and Welfare in 2011 [25]. The performance of our developed method was evaluated on wine samples spiked with target mycotoxins. Prior to the spiking, we analyzed the samples and confirmed that no mycotoxins were naturally present. The coefficient of linearity was determined using the samples spiked with each mycotoxin at the following levels: 5, 10, 20, 50, and 100 μg/L for PAT, NIV, DON, and ZON; 0.2, 0.5, 1, 2, and 5 μg/L for AFB1, B2, G1, G2, M1, and OTA; and 1, 2, 4, 10, and 20 μg/L for HT-2, T-2, FMB1, B2, and B3. Recovery and repeatability (relative standard deviation (%RSD)) experiments involved 5 replicate measurements that were carried out on the same day using the samples spiked with each mycotoxin at the following levels: 20 μg/L for PAT, NIV, DON, and ZON; 1 μg/L for AFB1, B2, G1, G2, M1, and OTA; 4 μg/L for HT-2 and T-2; and 5 μg/L for FMB1, B2, and B3.

## 3. Results and Discussion

### 3.1. Optimization of Clean-up and Analytical Conditions

**Figure 2:**
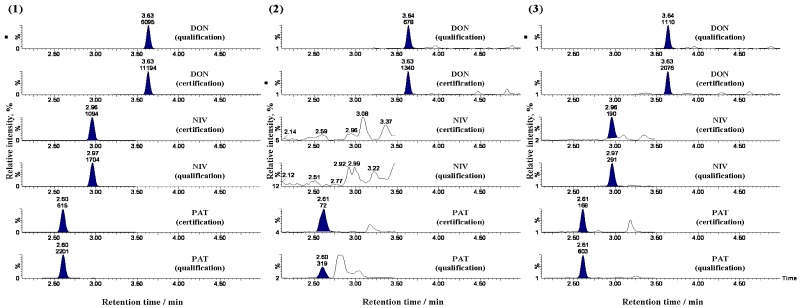
Chromatograms of patulin (PAT), NIV, and DON resulting from different pretreatment procedures. Each chromatogram was obtained from (**1**), the standards for PAT, NIV, and DON (each 20 μg/L); (**2**), the red wine samples spiked with mycotoxins (each 20 μg/L) that were extracted with a C18 cartridge after the QuEChERS approach; (**3**), the red wine samples spiked with mycotoxins (each 20 μg/L) that were extracted with a MultiSep #229 Ochra after the Oasis HLB cleanup.

**Figure 3:**
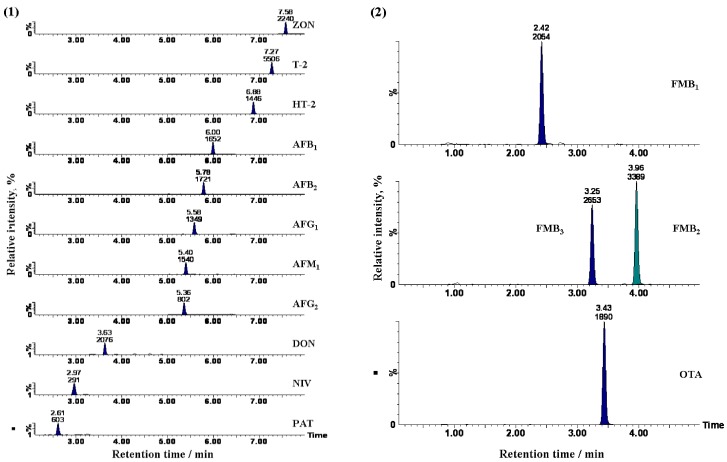
Ultra-high-performance liquid chromatography coupled to tandem mass spectrometry (UHPLC-MS/MS) chromatograms of the mycotoxins from extracted red wine samples.

The method that we previously reported for the analysis of beer-based drinks with the QuEChERS extraction [24], was applied first to red wine samples, and spiked tests were performed to optimize the procedure for extraction and clean-up of 15 mycotoxins, including NIV. In the UHPLC conditions, the column length and gradient profile have been changed from those in the previous study because it is assumed that matrix effects are more likely to occur when the mycotoxins are analyzed in wine than in beer-based drinks. The pigments were not sufficiently removed from the prepared samples, and injection of these samples may result in the lower analytical precision and UHPLC-MS/MS system contamination. Therefore, to sufficiently remove the pigments, the MultiSep #229 Ochra multifunctional cartridge was used instead of the C18 cartridge. Multifunctional cartridges have been widely adopted for the analysis of mycotoxins. They feature multiple solid supports for reverse-phase, normal-phase, cation exchange, anion exchange, or graphite carbon black, each of which can be used depending on the characteristic of target analytes; this allows for efficient removal of matrix components. Our previous study shows that several target mycotoxins were adsorbed on a primary-secondary amine and graphite carbon black [24]. A MultiSep #229 Ochra is designed to minimize adsorption of the target mycotoxins; hence, the clean-up procedure was examined using it. Pigments were removed from the red wines with this cartridge. However, on the chromatograms (Figure 2(2)), matrix peaks were observed proximally to PATs, and the PAT peaks were not as sharp as those on the standard chromatograms. No peaks were identified for NIVs. In either case, PAT and NIV peaks were affected by the presence of matrix components. Considering the retention time, it is assumed that these components were highly polar. Although this pretreatment combined extraction by acetonitorile and clean-up using the MultiSep #229 Ochra, it appeared difficult for this method to remove the highly polar matrices. Therefore, to remove such matrices, the sample was extracted and purified using the OasisHLB, rather than the QuEChERS methodology. An OasisHLB is a solid phase extraction cartridge that contains styrene-divinylbenzene co-polymer. It holds low to intermediate-polar substances with six-membered rings and separates highly polar substances. Eventually, nearly all of the pigments were removed from the wine samples that were purified with the multifunctional cartridge after being extracted and purified with the OasisHLB. No peaks for the highly polar matrices were observed, and the peak shapes for PAT and NIV were improved (Figure 2(3)). Using this clean-up method, pigments and highly polar matrices were removed from the wine samples, and good chromatograms were obtained. Figure 3 (1 and 2) shows UHPLC-MS/MS chromatograms of the purified red wine samples spiked with mycotoxins.

### 3.2. Performance Evaluation

Results of the evaluation are summarized in Table 2. Linearity of the calibration curves for the wine samples spiked with each mycotoxin were >0.990. Recovery rates ranged from 76 to 105% with the repeatability (as %RSD) ranging from 3.4 to 11.8% except for NIV. Recovery for NIV was 43%, which affected the quantification performance. It is assumed that the highly polar NIV was hardly held by the OasisHLB and was eluted with the matrices. Limits of quantification (LOQs) for the mycotoxins were the lowest concentration values shown on the calibration curves: 5 μg/L for PAT, DON, and ZON; 0.2 μg/L for AFB1, B2, G1, G2, M1, and OTA; and 1 μg/L for HT-2, T-2, FMB1, B2, and B3. 

Overall, we have successfully developed an accurate and sensitive method for the quantification of 14 mycotoxins, with the exception of NIV, in wine samples, and they were separated in a total of 13 min.

**Table 2:** Ultra-high-performance liquid chromatography (UHPLC)conditions for the determination of 14 mycotoxins.

Mycotoxin	Linearity ^1^	Repeatability (%) ^2^	Recovery (%) ^2^	LOQ (μg/L)	LOD (μg/L)	Retention time (min)
PAT	0.996	3.9	76	5	1.5	2.6
NIV	0.994	8.1	43	5	1.5	3.0
DON	0.999	7.6	96	5	1.5	3.6
AFG2	0.994	7.4	82	0.2	0.060	5.4
AFM1	0.994	5.7	94	0.2	0.060	5.4
AFG1	0.996	11.8	91	0.2	0.060	5.6
AFB2	0.994	9.4	90	0.2	0.060	5.8
AFB1	0.995	4.4	96	0.2	0.060	6.0
HT-2	0.999	5.2	99	1	0.30	6.9
T-2	0.999	3.4	93	1	0.30	7.3
ZON	>0.999	4.2	78	5	1.50	7.6
FMB1	0.999	4.1	76	1	0.30	2.4
FMB2	>0.999	6.0	82	1	0.30	4.0
FMB3	>0.999	5.1	94	1	0.30	3.3
OTA	0.990	8.6	105	0.2	0.06	3.4

^1^ Coefficient of linearity was determined using red wine samples spiked with each mycotoxin at the following levels: 5, 10, 20, 50, and 100 μg/L for PAT, NIV, DON, and ZON; 0.2, 0.5, 1, 2, and 5 μg/L for AFB1, B2, G1, G2, M1, and OTA; and 1, 2, 4, 10, and 20 μg/L for HT-2, T-2, FMB1, B2, and B3. ^2^ Recovery and repeatability [relative standard deviation (%RSD)] experiments involved 5 replicate measurements that were carried out on the same day using red wine samples spiked with each mycotoxin at the following levels: 20 μg/L for PAT, NIV, DON, and ZON; 1 μg/L for AFB1, B2, G1, G2, M1, and OTA; 4 μg/L for HT-2 and T-2; and 5 μg/L for FMB1, B2, and B3.

### 3.3. Analysis of Commercially Available Samples

Twenty-seven domestic and imported wines available in Japan were analyzed using our developed method. Results are summarized in Table 3. In none of the samples tested were any of the target mycotoxins detected, except for FMs and OTA.

**Table 3:** Mycotoxins detected in analyzed samples.

Sample	Concentration (μg/L) of mycotoxin				Sample	Concentration (μg/L) of mycotoxin			
	FMB1	FMB2	FMB3	OTA		FMB1	FMB2	FMB3	OTA
Red-1	<1.0	<1.0	<1.0	<0.20	White-1				0.42
Red-2	<1.0			<0.20	White-2	<1.0			
Red-3	<1.0				White-3	<1.0			
Red-4				0.20	White-4				
Red-5	<1.0			<0.20	White-5				
Red-6		<1.0			White-6				
Red-7					White-7				
Red-8					White-8				
Red-9					White-9				
Red-10					White-10				
Red-11					White-11				
Red-12					White-12				
Red-13					White-13				
Red-14									

The cells in the table without a value indicate no mycotoxins were detected.

FMs and/or OTA were detected in 6 of the red wine samples. All of the FMs detected were at values less than LOQ (1 μg/L). The maximum OTA value was 0.20 μg/L but this is less than its maximum level established by the EU (2 μg/L). This result indicates that the health risk posed to consumers from red wine is relatively low. However, it will be necessary to keep monitoring them in conjunction with other mycotoxins in the future because the co-occurrence of different FMs in 1 sample and the co-occurrence of FMs and OTA in 3 samples were observed. 

Moreover, FMB1 and OTA were detected in 2 and 1 samples of white wines, respectively; the co-occurrence of FMs and/or OTA was not observed in all white wine samples. This result clearly indicates that white wine samples are less subject to mycotoxin contamination in comparison with red wines.

As for FMs and OTA detected in wine samples, no regulatory levels have been set in Japan. Therefore, it is necessary to control risk of the contamination of mycotoxins and to closely watch the domestic and foreign trends.
